# B7-H4 is a potential diagnostic and prognostic biomarker in colorectal cancer and correlates with the epithelial-mesenchymal transition

**DOI:** 10.1186/s12885-022-10159-5

**Published:** 2022-10-10

**Authors:** Xiaotian Yan, Bo Hong, Jie Feng, Yuanqing Jin, Mengting Chen, Fugang Li, Yun Qian

**Affiliations:** 1 Department of Clinical Laboratory, Stomatology Hospital, School of Stomatology, Zhejiang University School of Medicine, Zhejiang Provincial Clinical Research Center for Oral Diseases, Key Laboratory of Oral Biomedical Research of Zhejiang Province, Cancer Center of Zhejiang University, 166 North Qiutao Road, Hangzhou, Zhejiang Province 310006 China; 2 Department of Pathology, The Second Affiliated Hospital, Zhejiang University School of Medicine, Hangzhou, 310009 Zhejiang Province China; 3 Department of Blood Transfusion, The Second Affiliated Hospital, Zhejiang University School of Medicine, Hangzhou, 310009 Zhejiang Province China; 4 Department of Clinical Laboratory, The Second Affiliated Hospital, Zhejiang University School of Medicine, Hangzhou, 310009 Zhejiang Province China; 5 School of Medical Technology and Information Engineering, Zhejiang Chinese Medical University, Hangzhou, 310053 Zhejiang Province China; 6 Shanghai Upper Bio Tech Pharma Company Limited, Shanghai, 201201 China

**Keywords:** Colorectal cancer, B7-H4, Prognosis, Immunohistochemistry, Epithelial-mesenchymal transition

## Abstract

**Background:**

As a negative co-stimulatory molecule of the B7 family, B7-H4 has recently attracted increased attention. However, the clinical value of B7-H4 in colorectal cancer (CRC) remains controversial and requires further investigation. This study aimed to investigate the role of B7-H4 in the clinical diagnosis and survival prognosis of CRC.

**Methods:**

The relationships between B7-H4 expression, immune cell infiltration, epithelial-mesenchymal transition (EMT), clinicopathological features, and survival prognosis were determined through the TCGA database and verified in a large CRC cohort (*n* = 1118).

**Results:**

The results showed the level of B7-H4 mRNA expression was significantly increased in the CRC tumor tissues compared with normal tissues (*P* <  0.001). Immunohistochemistry showed that B7-H4 protein expression was also up-regulated in CRC. The positive rate of B7-H4 in CRC tumor tissues was 76.38%, which was significantly higher than that in non-tumor tissues (*P* <  0.001). Overexpression of B7-H4 was positively correlated with lymph node metastasis, advanced TNM stage, and poor tumor differentiation (*P* = 0.012; 0.009; 0.014). Prognostic analysis showed high B7-H4 expression was associated with significantly shorter OS. Multivariate analysis demonstrated the risk of death in CRC patients with high B7-H4 expression is 1.487 times that of low B7-H4 expression. In addition, B7-H4 expression was negatively correlated with the epithelial marker E-cadherin (*P* <  0.001) and positively correlated with the mesenchymal marker vimentin (P <  0.001) in CRC tissues. However, B7-H4 expression was not associated with the immunosuppressive microenvironment in CRC.

**Conclusion:**

B7-H4 may represent a potential biomarker for the diagnosis and prognosis of CRC and enhance CRC invasion by promoting EMT.

**Supplementary Information:**

The online version contains supplementary material available at 10.1186/s12885-022-10159-5.

## Introduction

According to the Global Cancer Statistics report released by the International Agency for Research on Cancer (IARC) in 2020, colorectal cancer (CRC) was listed as the most common gastrointestinal malignant tumor, ranking third for morbidity and second for mortality [1]. In China, the incidence and mortality of CRC rank second, resulting in 560,000 new cases and 290,000 deaths in 2020 [2]. In addition, the lack of specific clinical symptoms and early screening tests, indicate that a portion of CRC patients is first diagnosed at an advanced stage, which leads to a poor prognosis. Therefore, CRC represents a serious burden on human health.

The conventional treatment for CRC consists of surgical resection, neoadjuvant chemotherapy, and targeted therapy. As the most promising treatment method, there has recently been increased use of immunotherapy in clinical trials. Programmed cell death protein 1 (PD-1)/programmed death-ligand 1 (PD-L1) pathway blockade showed effective and durable antitumor effects in a wide range of tumors [3]; however, its therapeutic effect in CRC is not as potent as in other tumors, and only a minority of patients benefit from approved checkpoint inhibitors [4]. Therefore, new immune suppression checkpoints must be identified to stimulate long-lasting anti-tumor immune responses in CRC patients.

As a negative co-stimulatory molecule of the B7 family, B7-H4 has recently attracted increased attention [5]. Moreover, studies have shown that B7-H4 promotes tumor progression by suppressing T cell immune responses. It has also been reported that B7-H4 has limited expression in normal tissues but is overexpressed in pancreatic cancer [6], ovarian cancer [7], breast cancer [8], and other malignant tumors. Several studies have suggested that B7-H4 can be used as a biomarker of malignant tumor progression and prognosis. However, the clinical significance and prognostic value of B7-H4 in CRC remains controversial and requires further investigation.

Epithelial-mesenchymal transition (EMT) refers to the process by which epithelial cells transform into a mesenchymal phenotype, which has been hypothesized to be a key event in promoting tumor invasion and metastasis [9, 10]. Throughout this process, the mesenchymal markers vimentin and N-cadherin are up-regulated, whilst the epithelial marker E-cadherin is down-regulated. Activation of EMT has recently been identified in several solid cancers, including CRC [11, 12]. New insights into anticancer strategies and therapeutic targets for CRC can be gained by exploring the molecular mechanisms involved in the regulation of EMT. As a potential candidate target for cancer therapy, B7-H4 may play a vital role in promoting the EMT process, which remains to be further elucidated.

In the present study, we investigated the clinical significance of B7-H4 in CRC and the relationship between B7-H4 expression and EMT status using The Cancer Genome Atlas (TCGA) database and verified this association in a large clinical CRC cohort. This study primarily analyzed the level of B7-H4 mRNA and protein expression with the aim of exploring the clinical significance and prognostic value of B7-H4 and providing potential clinical treatment strategies for CRC patients.

## Materials and methods

### Transcriptome data analysis in the TCGA and genotype-tissue expression (GTEx) databases

The transcriptome data and the corresponding clinical information of CRC tumor tissues from TCGA database and the matched normal colorectal tissues from the GTEx database were downloaded using the R language package. For further analysis, mRNA expression data were first normalized according to the requirements on the TCGA and GTEx websites. A total of 644 CRC tissues and 308 normal tissues were finally included and analyzed for B7-H4 mRNA expression.

### Immune infiltration analysis based on single-sample geneset enrichment analysis (ssGSEA)

ssGSEA is a deconvolution algorithm that evaluates the level of immune cell infiltration in a sample based on the expression level of immune cell-specific marker genes [13]. We performed ssGSEA analysis of the enrichment scores of tumor immune cells in Colon adenocarcinoma (COAD) and Rectum adenocarcinoma (READ) patients from the TCGA dataset, an approach we have used before [14].

The following 24 types of immune cells were obtained: activated dendritic cells (aDC), B cells, CD8+ T cells, Cytotoxic cells, dendritic cells (DC), eosinophils, immature dendritic cells (iDC), macrophages, mast cell, neutrophils, NK CD56 bright cells, NK CD56dim cells, natural killer cells (NK cells), plasmacytoid dendritic cells (pDC), T cells, T helper cells, central memory T cells (Tcm), effector memory T cells (Tem), follicular helper T cells (TFH), Tgd, type-1 T helper cells (Th1), type-17 T helper cells (Th17), type-2 T helper cells (Th2) and T regulatory cells (Tregs). Subpopulations of macrophages were further analyzed, including M1 and M2 macrophages.

### Gene set enrichment analysis (GSEA) of B7-H4 in CRC

With the help of the R software package “clusterProfiler”, we performed GSEA based on the Gene Ontology (GO), Kyoto Encyclopedia of Genes and Genomes (KEGG) [15–17], and Reactome datasets to explore the biological function of B7-H4 in tumor progression [18]. We used the “enrichplot” package to show the top five signaling pathways most significantly enrichment enriched in the database.

### Patients and tissue samples

The study followed up 1258 patients who underwent CRC surgery at the Second Affiliated Hospital of Zhejiang University School of Medicine from December 2002 to November 2011. CRC tissues and corresponding medical records were collected for all of the patients. The inclusion criteria consisted of 1) primary; 2) diagnosis of CRC by histopathology; 3) no radiotherapy/chemotherapy before surgery, and 4) complete clinical data. The exclusion criteria consisted of 1) patients with other infections or autoimmune diseases; 2) patients who suffer from other substantial diseases, and 3) patients who had undergone immunosuppressive therapy.

This study finally included a total of 1118 patients. The average follow-up time was 101 months (range: 1 - 227 months). This study followed the ethical guidelines of the Declaration of Helsinki and was approved by the Ethics Committee of the Second Affiliated Hospital of Zhejiang University School of Medicine.

### Tissue microarray

A tissue microarray (TMA) was performed by an experienced pathologist. The H&E slides were examined to locate both the tumor and adjacent non-tumor tissues (refers to the normal colonic mucosa adjacent to the tumor tissues). A manual tissue microarrayer was used to punch out a 1.0 mm diameter tissue cylinder from the representative tumor area of each tissue block and it was transferred into the hole of the prefabricated recipient paraffin block. The adjacent tissues were transferred into the holes of a paraffin block in the same manner. Each TMA was made in a corresponding arrangement of the tumor tissue-adjacent tissue. A total of 40 holes were placed on each TMA, making a total of 29 TMAs.

### Immunohistochemistry (IHC)

Formalin-fixed and paraffin-embedded TMA blocks were sectioned into 5-μm-thick pieces and subjected to IHC staining. The slides were deparaffinized with xylene and rehydrated through a series of successively increasing dilutions of alcohol. Endogenous peroxidase activity was blocked by using a 0.3% hydrogen peroxide solution at 25 °C for 30 min, and antigen retrieval was performed by boiling the sections at 100 °C for 30 min in citrate buffer (10 mmol/L; pH 6.0). Slides were washed 3 times with phosphate-buffered saline (PBS) for 5 min each, then incubated with 10% normal goat serum to block nonspecific binding. The sections were subsequently incubated with a rabbit anti-human B7-H4 monoclonal antibody (1:400 dilution; clone number EP1165; Abcam, MA, USA) at 4 °C overnight and a DAKO EnVision detection system (K5007, Dako, Glostrup, Denmark) was used to perform the immunoassay as previously described [14]. Slides were counterstained with Mayer’s hematoxylin, dehydrated with serial dilution of alcohol, and mounted with neutral resin.

TMAs were also stained with a mouse anti-human E-cadherin monoclonal antibody (1:200 dilution; clone number HECD-1; Abcam, MA, USA) and a rabbit anti-vimentin monoclonal antibody (1:500 dilution; clone number EPR3776; Abcam, MA, USA). Negative control staining was performed with PBS instead of a primary antibody. Human breast cancer and kidney tissues were stained as positive controls.

### Manual quantification of IHC. Quantification of IHC staining was performed by two pathologists blinded to the clinical characteristics

The percentage of B7-H4 positive expression was divided into five levels according to the positive cell rate: 0 (0%); 1 (1–10%); 2 (10–50%); 3 (50–70%); and 4 (70–100%). Based on the staining intensity, positive B7-H4 expression was divided into the following four grades: 0 (no staining); 1 (weak staining, light yellow); 2 (mild staining, yellow-brown); and 3 (strong staining, dark brown). Two indicators (staining intensity and percentage of positive cells) were combined to provide a semi-quantitative score, and the product of these two indicators was used to provide the final IHC score (0 to 12). Based on the IHC score, the tissue staining pattern was defined as either low expression (IHC score = 0–3) or high expression (IHC score = 4–12) [14].

The percentage of E-cadherin positive expression was divided into five levels according to the positive cell rate: 0 (0–5%); 1 (6–25%); 2 (26–50%); 3 (51–75%); and 4 (76–100%). Based on the staining intensity, positive E-cadherin expression was divided into the following four grades: 0 (no staining); 1 (weak staining, light yellow); 2 (mild staining, yellow-brown); and 3 (strong staining, dark brown). Two indicators were combined to provide a semi-quantitative score, and the product of these two indicators was used to provide the final IHC score (0 to 12). Based on the IHC score, the tissue staining pattern was defined as either negative (IHC score = 0–6) or positive (IHC score = 7–12) [19]. It should be noted that only membranous brown staining was considered positive for E-cadherin expression in any number of cells [20]. The expression was assessed only in tumor cells because it was difficult to assess membrane expression in stromal cells.

The staining of vimentin in tumor cells was scored according to the intensity [16]: 0 (no expression), 1 (fragmented membranous and/or weak to moderate expression), 2 (strongly fragmented or fully membranous/cytoplasmic moderate expression), and 3 (fully membranous/cytoplasmic strong expression). A score of < 2 was considered a negative vimentin expression, and ≥ 2 was considered a positive vimentin expression.

Patients considered to be EMT positive were those that were scored both vimentin ‘positive’ and E-cadherin ‘negative’.

### Statistical analysis

Statistical analysis and visualization were performed using Statistical Products and Services Solutions (SPSS) 22.0 statistical software and GraphPad Prism 9.0 software for this study. Statistical comparisons were assessed using the t-test, Spearman correlation analysis, chi-square test, and Fisher’s exact test as appropriate. Survival analysis was performed using the Kaplan-Meier (K-M) method and survival curves were compared using the log-rank test. *P* <  0.05 was considered to represent statistical significance.

## Results

### The level of B7-H4 mRNA expression in colorectal tumor tissues and corresponding normal tissues in the TCGA and GTEx databases

**Fig. 1:**
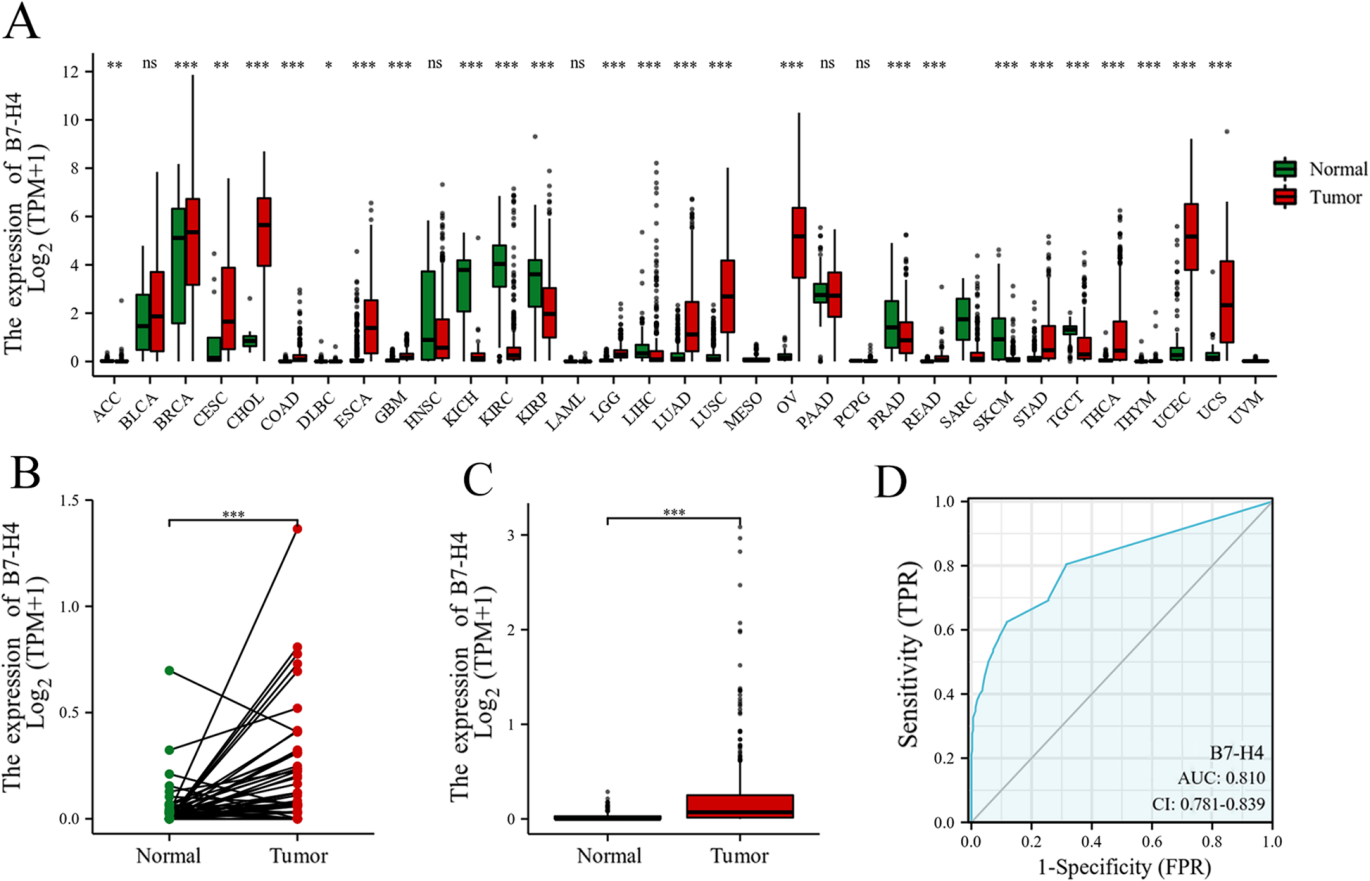
The expression level of B7-H4 mRNA in CRC patients from the TCGA and GTEx databases. **A** The expression of B7-H4 mRNA in human cancers from the TCGA database. **B** Paired sample analysis of B7-H4 mRNA expression levels in CRC patients in the TCGA database (*P* < 0.001). (**C**) The difference of B7-H4 mRNA expression between colorectal tumor tissue and normal tissue (*P* < 0.001) from the TCGA and GTEx databases. **D** The receiver operating characteristic (ROC) curve of B7-H4 mRNA expression for predicting the outcome

The level of B7-H4 mRNA expression in TCGA pan-cancer was analyzed, which was shown in Fig. 1A. It can be found that both COAD and READ showed higher expression of B7-H4 mRNA in tumor tissues than in normal tissues although the overall expression of B7-H4 mRNA was not high (both *P* <  0.001).

A paired sample comparison found that in CRC patients from the TCGA database, tumor tissues expressed higher levels of B7-H4 mRNA expression than adjacent non-tumor tissues (*P* < 0.001; Fig. 1B). The level of B7-H4 mRNA expression in 383 tumor tissues, 51 adjacent non-tumor tissues from the TCGA database, and 308 normal tissues from the GTEx database were also compared. We found that the tumor tissues of CRC patients exhibited significantly higher levels of B7-H4 mRNA expression compared to that of the normal tissues (*P* < 0.001; Fig. 1C).

### Correlation between B7-H4 mRNA expression and clinicopathological characteristics of the CRC patients from the TCGA database

The receiver operating characteristic (ROC) curve revealed that B7-H4 mRNA has superior diagnostic ability to distinguish between normal colonic mucosa and colorectal adenocarcinoma (Fig. 1D). The maximum area under the curve (AUC = 0.810) was obtained when the cut-off value of B7-H4 mRNA reached 0.033, which also showed a favorable balance between sensitivity (62.4%) and specificity (88.3%).

According to the B7-H4 mRNA cut-off value, a total of 642 CRC patients from the TCGA database were divided into low expression and high expression groups. The correlation between the level of B7-H4 mRNA expression and clinicopathological characteristics was analyzed (Table 1). The results showed that although CRC patients with high B7-H4 mRNA expression tended to have a more advanced TNM stage, no significant differences were observed between the high and low B7-H4 expression groups (*P* > 0.05). We found that CRC patients with low B7-H4 mRNA expression were more likely to develop neural invasion compared to those patients with high B7-H4 mRNA expression (*P* = 0.018).

**Table 1:** Correlation between B7-H4 mRNA expression and clinicopathological characteristics of CRC patients from the TCGA database

Characteristics	Total (N)	B7-H4 mRNA low expression	B7-H4 mRNA high expression	***P***-value
N	642	209	433	
Age (yr)				
≤ 60	197	56	141	0.137
> 60	445	153	292	
Gender				
Male	341	109	232	0.734
Female	301	100	201	
Tumor location				
Colon	478	152	326	0.486
Rectum	164	57	107	
Depth of tumor invasion				
T1/2	132	46	86	0.528
T3/4	510	163	347	
Lymph node metastasis				
N0	370	126	244	0.344
N1/2	272	83	189	
Distant metastasis				
No	542	178	364	0.310
Yes	91	25	66	
Not available	9	6	3	
TNM stage				
I/II	354	120	234	0.267
III/IV	279	83	196	
Not available	9	6	3	
Neural invasion				
Yes	60	27	33	0.018*
No	173	49	124	
Not available	409	133	276	
Vascular invasion				
Yes	135	41	94	0.411
No	424	145	279	
Not available	83	23	60	
Residual tumor				
R0	466	158	338	0.076
R1/R2	42	19	23	
Not available	134	32	72	
Recurrence				
Yes	125	34	91	0.155
No	517	175	342	
Alive	515	170	345	

*P < 0.05

### The prognostic value of B7-H4 mRNA in CRC patients from the TCGA database

**Fig. 2:**
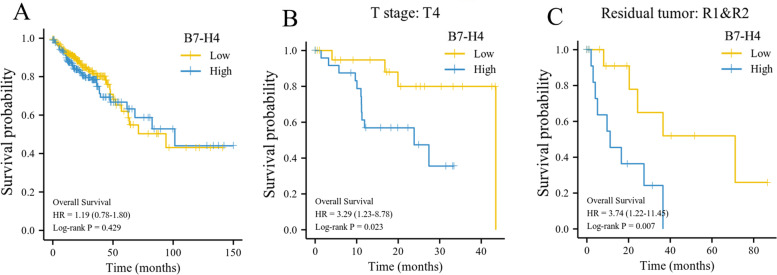
The survival analysis of B7-H4 mRNA in CRC patients from the TCGA database. **A** Survival analysis of CRC patients with high (yellow line) or low (blue line) B7-H4 expression (*P* = 0.429). **B** Survival analysis among CRC TNM Stage IV patients with high (yellow line) or low (blue line) B7-H4 expression (*P* = 0.023). **C** Survival analysis among CRC residual tumors (R1/R2) in patients with high (yellow line) or low (blue line) B7-H4 expression (*P* = 0.007)

The K-M survival analysis showed that while the CRC patients with low B7-H4 expression had a relatively short OS, no significant difference was observed between the B7-H4 high and low expression groups (*P* > 0.05; Fig. 2A). Further subgroup analysis revealed that among the TNM Stage IV patients, high B7-H4 mRNA expression was associated with a significantly shorter OS compared with the low B7-H4 mRNA expression group (*P* = 0.023; Fig. 2B). In patients with residual tumors (R1/R2), high B7-H4 mRNA was associated with a shorter OS (*P* = 0.007; Fig. 2C). These findings suggest that high B7-H4 mRNA expression may be an indicator of poor prognosis in malignant patients.

### Relationship between B7-H4 mRNA expression and tumor-infiltrating immune cells

**Fig. 3:**
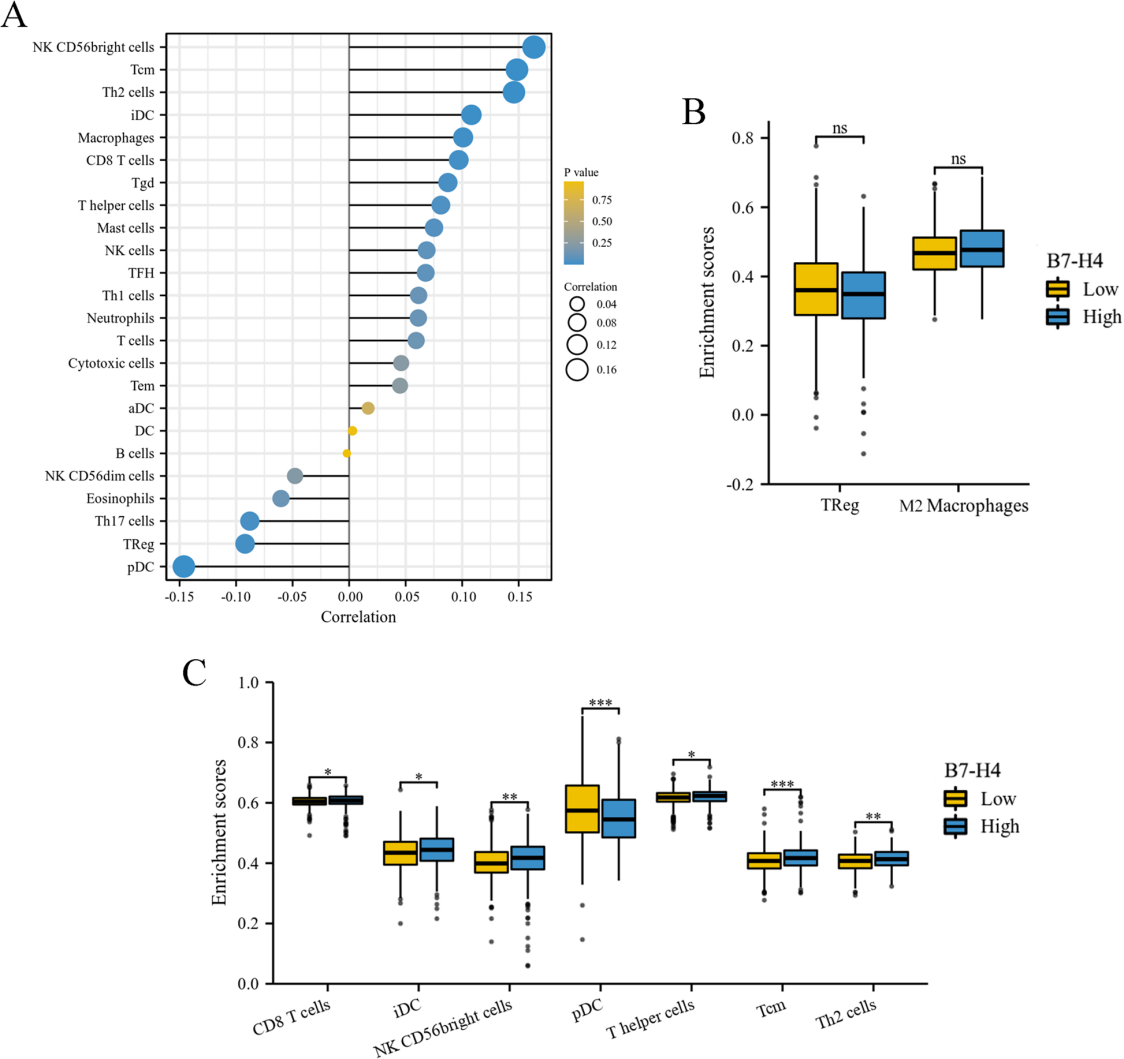
Relationship between B7-H4 mRNA expression and tumor-infiltrating immune cells. **A** The correlation between the expression of B7-H4 mRNA and 24 types of tumor-infiltrating immune cells. **B** 7 types of immune cells were significantly associated with B7-H4 mRNA expression (*P* < 0.05). (C) M2 macrophages and Treg cells were not significantly associated with B7-H4 mRNA expression (*P* > 0.05)

The correlation between 24 tumor-infiltrating immune cells and B7-H4 mRNA expression was shown in Fig. 3A. It can be found that NK CD56bright cells, Tcm, Th2 cells, iDC, Macrophages, CD8+ T cells, Tgd, T helper cells, Mast cells, NK cells, TFH, Th1 cells, neutrophils, T cells, cytotoxic cells, Tem, aDC and DC were positively correlated with B7-H4 mRNA expression. B cells, NK CD56dim cells, eosinophils, Th17 cells, Treg and pDC were negatively correlated with B7-H4 mRNA expression.

The distribution of tumor-infiltrating immune cells in the high and low expression groups of B7-H4 was significantly different in CRC, which was shown in Fig. 3B. Particularly, patients with high B7-H4 expression had higher CD8+ T cells, iDC, NK CD56bright cells, T helper cells, Tcm, and Th2 cells (*P* < 0.05). In contrast, patients with low B7-H4 expression had higher pDC (P < 0.05). In addition, we focused on two representative immunosuppressive cells, M2 macrophages, and Treg cells, and found that they were not associated with the expression of B7-H4 (Fig. 3C, *P* > 0.05).

Arguably, patients with high expression of B7-H4 mRNA tend to exhibit a subtle immune-activated status.

### Relationship between B7-H4 mRNA expression and EMT status

**Fig. 4:**
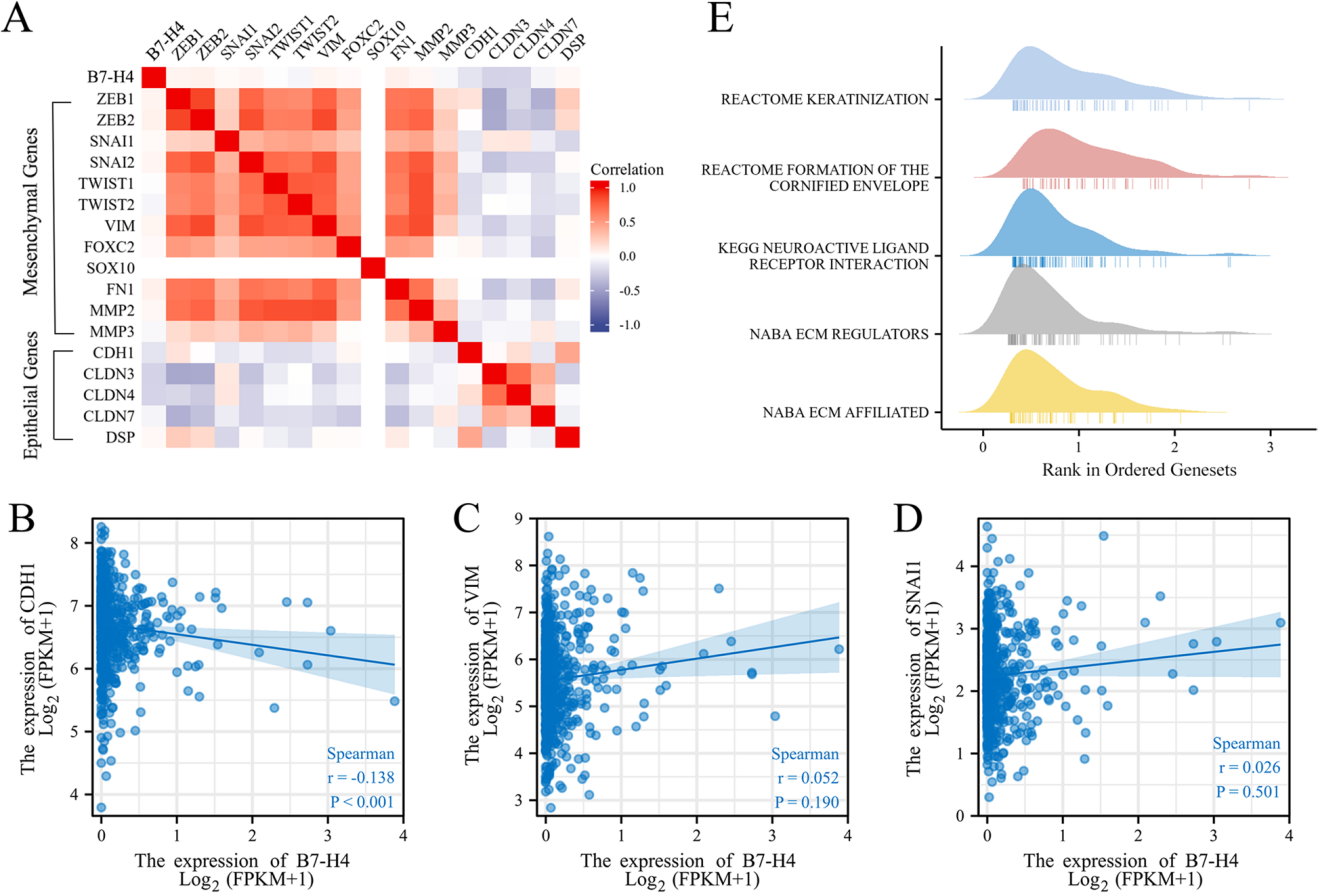
Relationship between B7-H4 mRNA expression and EMT status. A Heat map showing the mRNA expression level of B7-H4 between mesenchymal genes and epithelial genes in TCGA database. **B**-**D** Scatter plots showing the correlation of B7-H4 and EMT-related genes (CDH1, VIM, and SNAI1) expression in CRC samples from the TCGA database. **E** Ridgeline plot for B7-H4 related enriched gene sets analyzed by GSEA

In the TCGA CRC patient’s cohort, the correlation of B7-H4 gene expression with mesenchymal and epithelial genes expression was analyzed. Heatmap showed that B7-H4 mRNA was on the whole positively correlated with the mRNA expression of mesenchymal genes and negatively correlated with the mRNA expression of epithelial genes (Fig. 4. A).

Among them, we mainly focus on the classic markers involved in the EMT process, such as E-cadherin (gene name CDH1), vimentin (gene name VIM), and snail (gene name SNAI1). Spearman correlation analysis showed that the expression of B7-H4 was significantly negatively correlated with the expression of E-cadherin (*R* = − 0.138, *P* < 0.001, Fig. 4. B) in CRC samples, and the expression of B7-H4 was positively correlated with the expression of vimentin and snail, but not significant (*R* = 0.052, 0.026, both *P* > 0.05, Fig. 4. C-D).

Furthermore, in order to observe the enrichment status of B7-H4 in the cancer pathway, we performed a GSEA by comparing transcriptomes between cases with high and low B7-H4 expression. The top five signaling pathways with the most significant enrichment were listed in Fig. 4E. It was found that keratinization, formation of the cornified envelope, neuroactive ligand-receptor interaction, extracellular matrix (ECM) regulators, and ECM affiliated were described as the most abundant pathways in CRC patients with high B7-H4 expression. This suggested that B7-H4 showed a strong relationship with epithelial cell keratinization and regulation of ECM components, further reminding us that B7-H4 may promote the EMT process by controlling cell differentiation and development.

### B7-H4 protein expression pattern in a large CRC cohort

We collected a total of 1118 cases of CRC tumor tissues and 912 cases of adjacent non-tumor tissues (part of the tissues were discarded due to errors in the extraction or film production process). After IHC staining and semi-quantitative scoring, the positive rate of B7-H4 protein expression in the tumor cells of CRC patients was 76.38%, of which high expression accounted for 61.0% and low expression accounted for 39.0%. The positive rate of B7-H4 protein expression in the tumor stroma of CRC patients was 20.13%, and almost all of them were low expression. Due to the expression of B7-H4 in tumor stroma was rather low, only the expression of B7-H4 in tumor cells was analyzed subsequently. The level of B7-H4 protein expression in the paired adjacent normal colorectal tissues indicated that the positive rate was 49.89%, of which high expression accounted for 34.4% and the low expression accounted for 65.6%. However, the level of B7-H4 expression in the tumor tissues was significantly higher than that in the adjacent non-tumor tissues (Table 2; *P* < 0.001).

**Table 2:** Summary of B7-H4 expression in colorectal cancer and adjacent non-tumor tissues

	Colorectal Cancer Tissues	Adjacent Non-tumor Tissues	***P***-value
N	1118	912	
Positive Rate	76.38%	49.89%	
Low expression (%)	436 (39.0%)	598 (65.6%)	< 0.001
High expression (%)	682 (61.0%)	314 (34.4%)	

**Fig. 5:**
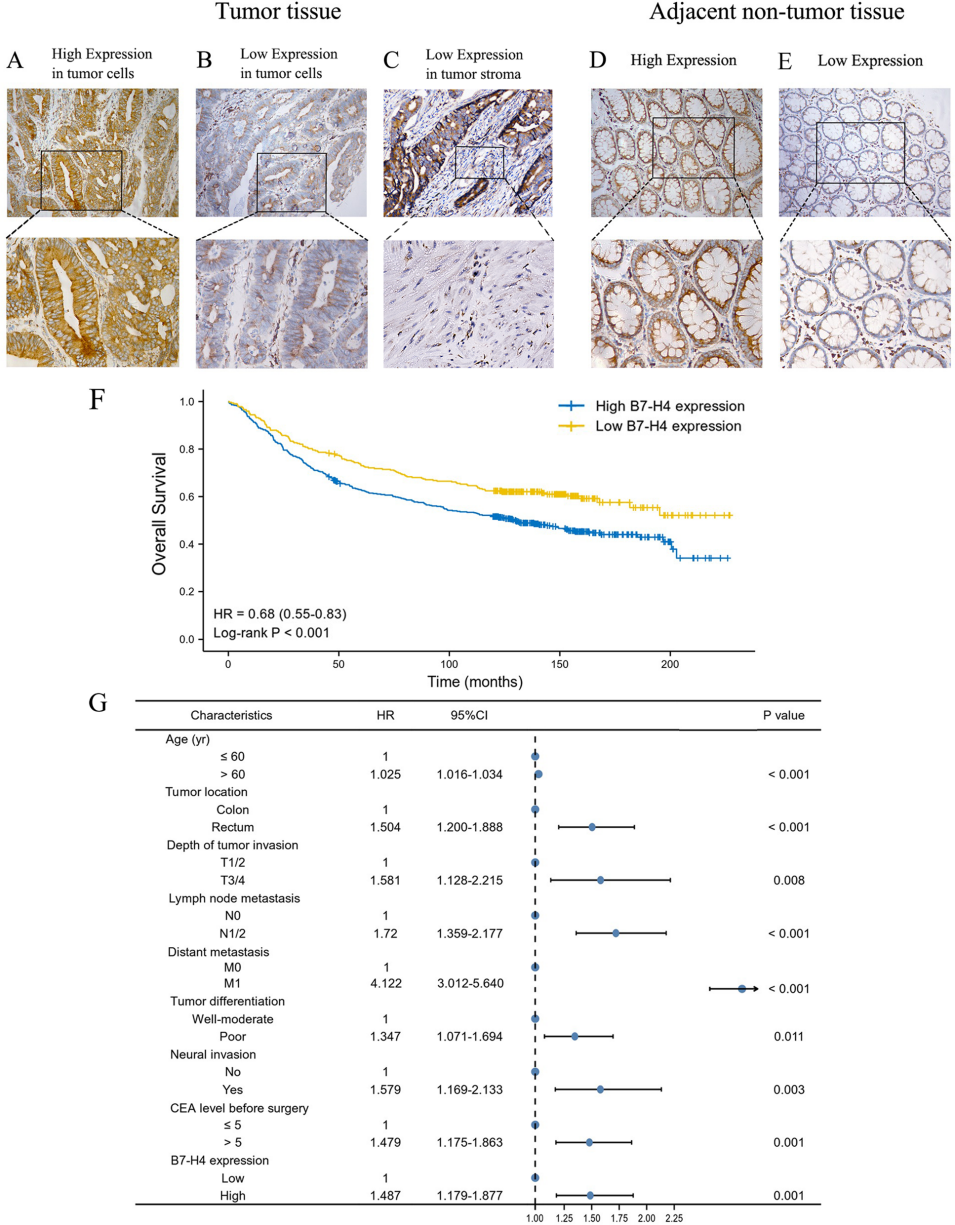
The expression pattern and survival analysis of B7-H4 in clinical cohort of CRC patients. **A**-**E** Representative staining images of B7-H4 in CRC tumor cells and stroma (**A**-**C**) and adjacent non-tumor tissues (**D**-**E**). Original magnification × 200 and × 400. (**F**) The K-M curve between CRC patients with high (blue line) or low (yellow line) B7-H4 expression (*P* < 0.001). (**G**) Multivariate Cox proportional hazard analysis of the risk factors correlated with overall survival. Hazard ratios with 95% confidence intervals of risk factors associated with CRC are provided (both *P* < 0.01)

B7-H4 protein expression patterns in CRC tumor cells and stroma appeared to be diffuse in most cases. Under the microscope, B7-H4 was mainly observed in the tumor cell membrane, cytoplasm, or both, and occasionally observed in the tumor stroma. In the adjacent non-tumor tissues, B7-H4 was primarily expressed in the cytoplasm of glandular cells. Representative images are shown in Fig. 5A-E. In addition, we investigated the B7-H4 expression pattern based on the distribution of B7-H4 in tumor tissues and adjacent non-tumor tissues. It was found that B7-H4 was highly expressed in the tumor area but expressed at low levels in the adjacent non-tumor area in the majority of CRC cases (Table S1).

### Statistical association between B7-H4 and clinicopathologic features of CRC patients in the clinical cohort

The correlation between clinicopathological characteristics and B7-H4 protein expression was analyzed in 1118 CRC primary tumors, as shown in Table 2. Compared to patients with low B7-H4 expression, patients with high B7-H4 expression were more likely to exhibit lymph node metastasis (Table 3; *P* = 0.012) and had a worse degree of tumor differentiation and TNM stage (Table 3, *P* = 0.009 and *P* = 0.014, respectively). No other significant differences were observed regarding the association between B7-H4 and clinicopathological characteristics. To a certain extent, these results indicated that the overexpression of B7-H4 is closely associated with an aggressive phenotype, thereby implying an important role of B7-H4 in the progression of CRC.

**Table 3:** Association between clinicopathological features and B7-H4 expression in the primary tumors of CRC patients

Characteristics	Total (N)	B7-H4 low expression	B7-H4 high expression	***P***-value
N	1118	436	682	
Age (yr)				
≤ 60	527	217	310	0.159
> 60	591	219	372	
Gender				
Male	669	269	400	0.311
Female	449	167	282	
Family history				
Yes	54	21	33	0.987
No	1064	415	649	
Tumor location				
Colon	632	258	374	0.154
Rectum	486	178	308	
Tumor size (cm)				
≤ 5	825	321	504	0.918
> 5	293	115	178	
Depth of tumor invasion				
T1/2	221	88	133	0.78
T3/4	897	348	549	
Lymph node metastasis				
N0	630	266	364	0.012*
N1/2	488	170	318	
Distant metastasis				
Yes	97	33	64	0.293
No	1021	403	618	
TNM stage				
I/II	608	257	351	0.014*
III/IV	510	179	331	
Neural invasion				
Yes	71	25	46	0.499
No	1047	411	636	
Vascular invasion				
Yes	114	46	68	0.755
No	1004	390	614	
Mucinous adenocarcinoma				
Yes	221	97	124	0.096
No	897	339	558	
Tumor differentiation				
Poor	573	256	377	0.009**
Well-moderate	485	180	305	
Adjuvant treatment				
Yes	530	205	325	0.836
No	558	231	357	
CEA level before surgery				
≤ 5	593	228	365	0.639
> 5	345	138	207	

CEA levels before surgery were not detected in some patients, resulting in missing CEA data. *P < 0.05; **P < 0.01

### The prognostic value of B7-H4 protein expression in the follow-up CRC patients

Until October 2021, patients were followed up for 19 years, with an average follow-up time of 101 months (range: 1–227 months). Using the date of resection as the starting point, the end of OS was defined as the day when survival or death was confirmed. The K-M survival analysis was used to evaluate the prognostic value of B7-H4. Figure 5F shows that the levels of B7-H4 protein expression were related to CRC patient prognosis. High B7-H4 expression was associated with a significantly shorter OS compared to that of patients with low B7-H4 expression (*P* < 0.001).

The Cox proportional hazard ratio model was used to explore factors that may affect the OS of CRC patients. The multivariate analysis showed that age, tumor location, depth of tumor invasion, lymph node metastasis, distant metastasis, neural invasion, and CEA level before surgery and B7-H4 expression were independent prognostic factors for CRC patients. It is important to note that the risk of death in CRC patients with high B7-H4 expression is 1.487 times that of patients with low B7-H4 expression (Fig. 5G). These results indicated that the high expression of B7-H4 levels in CRC might help predict the poor prognosis of patients with CRC and inspire novel therapeutic strategies.

### Relationship between B7-H4 and EMT-correlated proteins expression

**Fig. 6:**
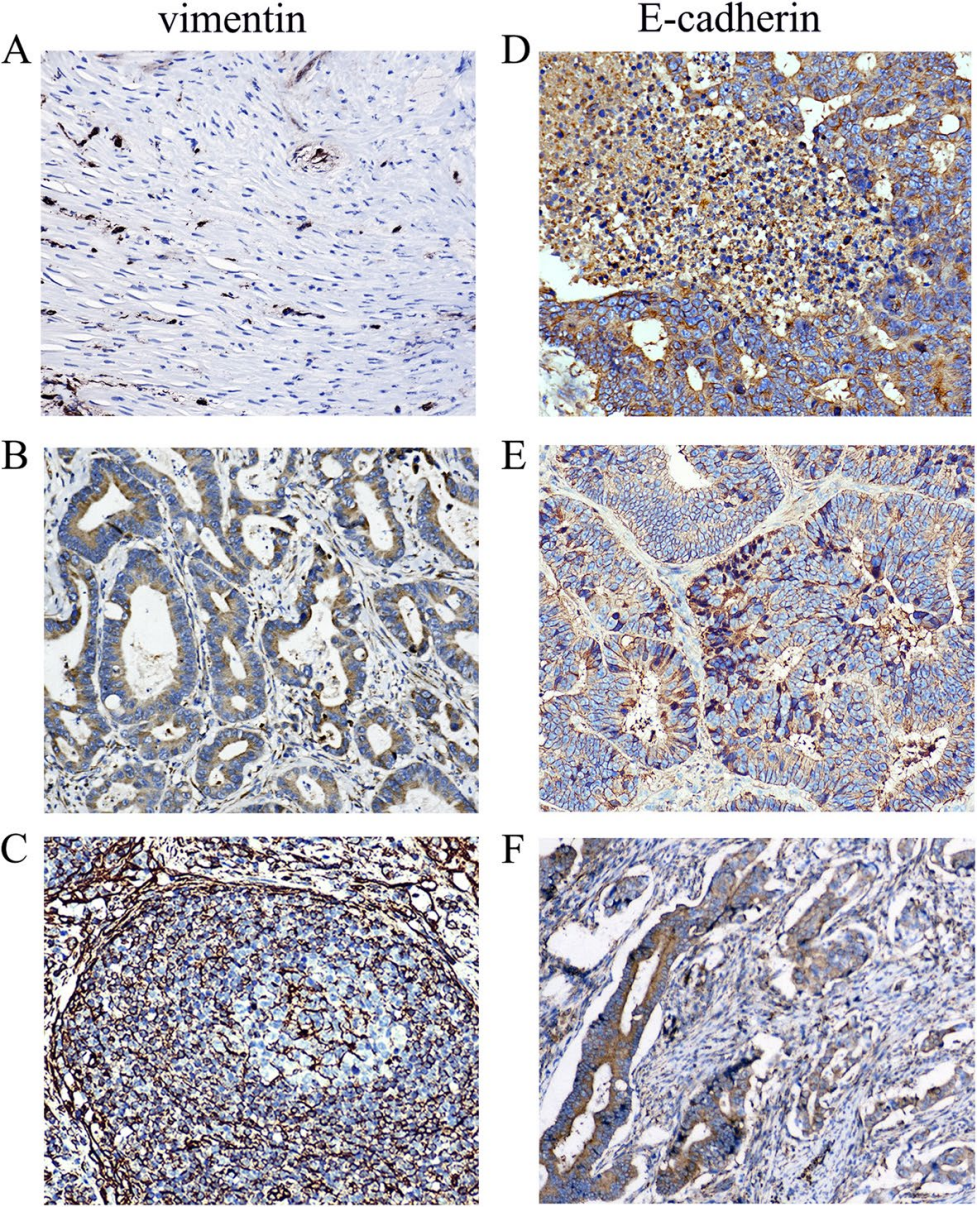
IHC staining for vimentin and E-cadherin in CRC tumor tissues. Representative staining images of vimentin and E-cadherin expression in CRC tumor tissue. **A** Mild vimentin expression in tumor stroma; **B** Moderate vimentin expression in tumor cells; **C** Strong vimentin expression in tumor cells; **D** Strong E-cadherin expression in tumor cells; (**E**) Moderate E-cadherin expression in tumor cells and mild expression in tumor stroma; **F** Mild E-cadherin expression in tumor cells and tumor stroma. Original magnification × 200

To investigate how B7-H4 affects CRC progression, we further analyzed the expression of B7-H4 and EMT-correlated proteins, such as the epithelial marker E-cadherin and the mesenchymal marker vimentin, in CRC tissue samples using IHC staining (Fig. 6). Since the TMA collected from 2002 to 2007 was not enough for redundant IHC staining, we concentratedly selected 451 patients from 2008 to 2011 for IHC staining of E-cadherin and vimentin. The results of IHC staining showed that E-cadherin was mainly expressed in the cell membrane and/or cytoplasm of tumor cells, and slightly expressed in the tumor stroma, while vimentin was mainly expressed in the membrane of tumor cells and rarely expressed in the tumor stroma.

The expression of B7-H4 was negatively correlated with E-cadherin (*P* < 0.001) and positively correlated with vimentin (*P* < 0.001) in CRC tissues (Table 4). Overall, patients with high B7-H4 expression were more likely to develop a positive EMT status (*P* < 0.001). These results indicated that overexpression of B7-H4 enhanced CRC tumor invasion by promoting EMT.

**Table 4:** The association between B7-H4 and EMT-correlated proteins expression in CRC

Variables	N	B7-H4 low expression	B7-H4 high expression	χ2	***P***
E-cadherin					
Negative	256	78 (38.81%)	178 (70.92%)	46.756	< 0.001
Positive	196	123 (61.19%)	73 (29.08%)		
Vimentin					
Negative	281	161 (80.10%)	120 (47.81%)	49.484	< 0.001
Positive	171	40 (19.90%)	131 (52.19%)		
EMT status					
Negative (E-cadherin+ vimentin-)	303	170 (84.58%)	133 (52.99%)	50.403	< 0.001
Positive (E-cadherin- vimentin+)	149	31 (15.42%)	118 (47.01%)		

## Discussion

Recent research on immune suppression checkpoints, including B7-H4, has gained popularity. Under normal circumstances, human B7-H4 mRNA is commonly expressed in normal tissues; however, B7-H4 protein exhibits minimal expression in normal tissues [21]. In contrast, abnormal B7-H4 expression has been observed in many tumor tissues. These findings indicate that normal tissues can strictly regulate the expression of B7-H4 at the post-transcriptional level, whereas this regulatory mechanism is inhibited or altered within the tumor environment, consistent with the findings of Yi KH et al. [22]. Moreover, clinical studies have shown that the expression of B7-H4 is significantly increased in ovarian, pancreatic, and breast cancer, and the expression level is related to advanced clinical stage and poor prognosis [6, 23–26]. Although few studies have focused on the role of B7-H4 in CRC tumor progression, the clinical significance of B7-H4 remains controversial and requires further investigation.

Bioinformatics analyses can provide a deeper and more comprehensive understanding of genes, allowing researchers to further analyze identified functional genes [27]. The TCGA database is an important resource for bioinformatics analyses as it provides researchers with various genetic information on the different types of cancer. With the help of the TCGA database, we found that B7-H4 mRNA was significantly up-regulated in CRC patients. In addition, survival analysis revealed that high B7-H4 mRNA expression in patients with higher malignancy (TNM IV or R1/R2) predicted a worse prognosis (*P* < 0.05). These results encouraged us to follow up with clinical validation of B7-H4.

A growing number of studies have shown that the characteristics of tumor-infiltrating immune cells in the tumor microenvironment are related to the occurrence and progression of cancer [28, 29]. As an important co-stimulatory molecule, B7-H4 mediates tumor immune escape by indicating T cell responses [29]. Thus, in most solid tumors, B7-H4 expression was found to be inversely correlated with the density of stromal Tumor-Infiltrating Lymphocytes and CD8+ T lymphocytes. Interestingly, our study found the opposite results. We found that B7-H4 positively correlated with CD8+ T cells and negatively correlated with two types of immunosuppressive cells, M2 macrophages, and Treg cells. We speculated that there may be two reasons for this: 1) The overall expression of B7-H4 mRNA in CRC is relatively low compared to other immunosuppressive molecules, and its immunosuppressive effect is not obvious. Whether this slight immune activation is further inhibited by B7-H4 overexpression remains unclear, and future work should be conducted to explore the mechanism. 2) Multiple immunosuppressive molecules are acting in the CRC tumor microenvironment, and B7-H4 may not be the “key molecule” that mainly regulates the immunosuppressive microenvironment in CRC. Although a clinical trial (NCT03514121) has found B7-H4 to be safe in targeted immunotherapy for the treatment of solid tumors [30], no clinical trials of B7-H4 in the CRC have been conducted. Our preliminary study suggests that the ability of B7-H4 for targeted treatment of CRC remains to be considered.

Further, we analyzed the expression pattern of B7-H4 in CRC at the protein level in a large CRC clinical cohort. Our results confirmed that the level of B7-H4 expression was significantly increased in the tumor tissue and was positively correlated with lymph node metastasis, advanced TNM stage, and poor tumor differentiation. We also found that high B7-H4 expression was an independent risk factor for poor prognosis in CRC patients. Taken together, we suggest that abnormal B7-H4 expression is involved in the malignant progression of CRC and can be used as a potential clinical monitoring indicator of CRC.

This was the first study to describe the pattern of B7-H4 expression in the tumor tissues and normal colonic mucosa of CRC patients. In analyzing the B7-H4 expression patterns in cancer and adjacent non-tumor tissues, it was found that most CRCs exhibit high B7-H4 expression in the cancer tissues and low B7-H4 expression in the adjacent non-tumor tissues. Although B7-H4 protein is expressed at extremely low levels in normal tissues, this study revealed different levels of B7-H4 protein expression were observed in the CRC adjacent non-tumor tissues in this study. This finding indicates that B7-H4 may serve as a predictor of cancer progression. In the future, it may be possible to reveal the progressive development of B7-H4 by analyzing CRC tumor tissues, adjacent non-tumor tissue, and normal colorectal tissue.

Although several studies have confirmed the high expression of B7-H4 in CRC, its prognostic significance is still controversial. Compared with other studies with shorter follow-ups and fewer patients, our research has more reference value and practical significance. This study was associated with some limitations. Firstly, although we included as many patients as possible to achieve clinical relevance, this study was a retrospective single-center study, which may still have selection bias. Therefore, a larger-scale prospective survey is required. Second, TMA construction using a single small core may not be representative, especially if the protein of interest exhibits significant heterogeneity in expression. However, an increasing number of studies have measured immune checkpoint expression in different tumors using TMAs with consistent results, demonstrating the reliability of this approach [31, 32]. In this study, 100 cases of colorectal cancer tissue chips were randomly selected from 1118 cases, and the corresponding whole tissue sections were performed for IHC staining, which confirmed the high consistency of the two methods, thus verifying the reliability of TMA IHC results in our study. Representative images are shown in Fig. S1.

Using GSEA enrichment analysis, we were surprised to find that the most abundant signaling pathway in CRC patients with high B7-H4 expression was keratinization and cornified envelope formation. Few studies have demonstrated a definite association between keratinization and cornified envelope formation and colorectal cancer progression, but through an extensive literature search, we found that keratinization is involved in the metastasis and invasion of cancer cells in addition to regulating a variety of biological processes including cell proliferation and growth, immune responses, and differentiation of skin appendages [33–36]. Several studies have shown that the expression of keratins, such as KRT17 and KRT15, is higher in colon cancer tissues than in normal colonic epithelial tissues, and increased concomitantly as the grade of T staging progresses [37, 38]. Previous studies have also suggested that B7-H1/PD-1-mediated regulation may be involved in the pathogenesis of multiple T cell-mediated inflammatory epithelial lesions [39]. Therefore, we hypothesized that the high expression of B7-H4 may mediate the immune response of intestinal epithelial cells, activate keratinization and formation of the cornified envelope, and promote the invasion and metastasis of colorectal cancer. This finding also strengthens our belief that B7-H4 plays a non-negligible role in EMT.

Previous studies have shown that overexpression of B7-H4 in cholangiocarcinoma promotes tumor progression through EMT [40]. In vitro experiment by our group demonstrated that the inhibition of B7-H4 increased cell-cell adhesion, decreased the formation of pseudopodia, increased the expression of E-cadherin, and decreased the expression of vimentin and CD44 in pancreatic cancer [41]. Moreover, our results indicated that B7-H4 also plays a role in the EMT process of CRC, reducing cell-cell contact and promoting tumor invasion by reducing E-cadherin expression and increasing vimentin expression.

In conclusion, the findings of this study revealed that B7-H4 expression is up-regulated in CRC, at both the mRNA and protein levels. Importantly, overexpression of B7-H4 in tumor tissues was positively correlated with malignant phenotypes and poor prognosis in patients with CRC. Moreover, B7-H4 expression played a vital role in the EMT process, correlating with the down-regulated expression of E-cadherin and up-regulated expression of vimentin. Since CRC carcinogenesis and development are multistep processes, they are caused by the cumulative effects of multiple molecules [42]. Our current work has only explored the potential clinical significance of one of these molecules. We look forward to exploring the combined effect of multiple molecules on the occurrence and development of CRC in the future. In addition, as an important negative co-stimulatory molecule in CRC, B7-H4 should be further studied in an effort to clarify its molecular mechanism to provide further supporting evidence for its application as a diagnostic marker in CRC. Future studies involving authoritative and larger sample sizes should be performed to confirm these conclusions.

## Supplementary Information

**Additional file 1: Fig. S1.** Paired IHC staining of B7-H4 in whole tissue sections and corresponding tissue microarrays of tumor tissues. (A-B)Both high expression in tumor cells; (C-D) Both low expression in tumor stroma; (E-F) Both moderate expression in tumor stroma; (G-H) Both low expression in tumor cells; (I-J) Both high expression in tumor cells and moderate expression in tumor stroma; (K-L)Both high expression in tumor cells; (M-N) Both high expression in tumor cells; (O-P) Both low expression in tumor cells.

## Data Availability

All data generated or analyzed during this study are included in this published article.

## Abbreviations

aDC: Activated dendritic cells
CRC: Colorectal cancer
COAD: Colon adenocarcinoma
DC: Dendritic cells
EMT: Epithelial-mesenchymal transition
ECM: Extracellular matrix
GO: Gene Ontology
GTEx: Genotype-Tissue Expression
GSEA: Gene set enrichment analysis
iDC: Immature dendritic cells
IHC: Immunohistochemistry
IARC: International Agency for Research on Cancer
K-M: Kaplan-Meier
KEGG: Kyoto Encyclopedia of Genes and Genomes
NK cells: Natural killer cells
OS: Overall survival
PBS: Phosphate-buffered saline
PFS: Progression-free survival
PD-1: Programmed cell death protein 1
PD-L1: Programmed death-ligand 1
pDC: Plasmacytoid dendritic cells
READ: Rectum adenocarcinoma
ssGSEA: Single-sample geneset enrichment analysis
SPSS: Statistical product and service solutions
TCGA: The Cancer Genome Atlas
TNM: Tumor node metastasis
Tcm: Central memory T cells
Tem: Effector memory T cells
TFH: Follicular helper T cells
Th1: Type-1 T helper cells
Th17: Type-17 T helper cells
Th2: Type-2 T helper cells
Tregs: T regulatory cells
TMA: Tissue microarray
